# Accurate somatic variant detection using weakly supervised deep learning

**DOI:** 10.1038/s41467-022-31765-8

**Published:** 2022-07-22

**Authors:** Kiran Krishnamachari, Dylan Lu, Alexander Swift-Scott, Anuar Yeraliyev, Kayla Lee, Weitai Huang, Sim Ngak Leng, Anders Jacobsen Skanderup

**Affiliations:** 1 Department of Computational and Systems Biology, Agency for Science Technology and Research, Genome Institute of Singapore, Singapore, Singapore; 2 School of Computing, National University of Singapore, Singapore, Singapore

**Keywords:** Machine learning, Cancer genomics, Cancer genomics, Software

## Abstract

Identification of somatic mutations in tumor samples is commonly based on statistical methods in combination with heuristic filters. Here we develop VarNet, an end-to-end deep learning approach for identification of somatic variants from aligned tumor and matched normal DNA reads. VarNet is trained using image representations of 4.6 million high-confidence somatic variants annotated in 356 tumor whole genomes. We benchmark VarNet across a range of publicly available datasets, demonstrating performance often exceeding current state-of-the-art methods. Overall, our results demonstrate how a scalable deep learning approach could augment and potentially supplant human engineered features and heuristic filters in somatic variant calling.

## Abstract (web summary)

Deep learning could be applied to the challenge of somatic variant calling in cancer by making use of large-scale genomic data. Here, the authors develop VarNet, a weakly supervised deep learning model for somatic variant calling in cancer with robust performance across multiple cancer genomics datasets.

## Introduction

Identification of somatic mutations from DNA sequencing of tumor samples is key to cancer research and the implementation of precision oncology. The process of calling mutations in tumor DNA is convoluted by both biological variation (e.g. tumor heterogeneity) and technical noise (e.g. sequencing errors) in the samples. Existing best-in-class methods for somatic variant calling commonly rely on statistical models of variant allele frequencies in combination with a series of heuristic filters to remove false positives^1,2^. Importantly, these methods have been developed through human expert knowledge of DNA-sequencing data and tumor biology.

Machine learning offers a complementary data-centric approach that can exploit the vast amounts of next-generation sequencing data generated today. For example, Strelka2^2^ supplements its probabilistic variant model with a machine learning model, which uses variant quality features to predict an aggregate confidence score for each candidate variant. SMuRF^3^ is an ensemble somatic variant caller that uses machine learning and variant features from four distinct variant callers to predict high-confidence variants. Neusomatic^4^ uses a deep learning model to make somatic variant predictions from the aggregated base and read counts in a small neighborhood around candidate variant sites. DeepVariant^5^, a germline variant caller, uses images of aligned DNA reads in combination with a deep learning model to predict variants, mimicking how human experts perform a manual review of candidate variants^6^. Intriguingly, deep learning models operating on raw DNA read alignments may learn rich representations of reads comprising both their complex interdependencies as well as the sequence context around mutated sites. However, this concept has not been explored for somatic variant calling where variants have to be evaluated in the context of deeper tumor sequencing data, intratumor heterogeneity, and matched normal reads.

Here we describe VarNet, which uses deep learning models trained on large amounts of tumor-sequencing data to predict somatic single nucleotide variants (SNV), insertions, and deletions (indels). VarNet creates image representations of aligned reads from tumors and matched normal genomes including their properties such as base quality, mapping quality, and strand bias. As supervised deep learning requires access to large labeled datasets, which are typically scarce and expensive to generate in cancer genomics, VarNet uses a weakly supervised learning approach where high confidence pseudo-labels are generated across 7 cancer types and more than 300 cancer whole genomes. We evaluate the performance of VarNet on both real and synthetic tumor benchmark datasets, demonstrating consistent performance often exceeding existing methods.

## Results

### Overview of approach

**Fig. 1:**
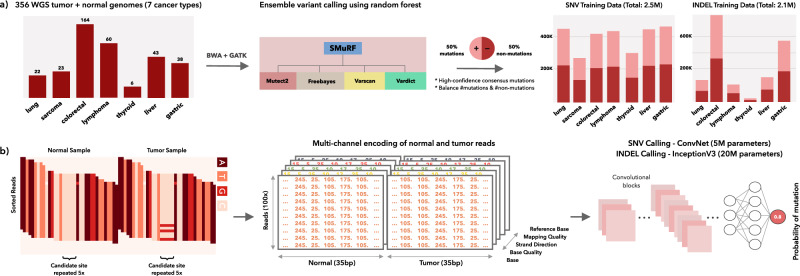
Overview of approach. **a** Matched tumor/normal genomes were used to generate training data. Training labels were generated using high-confidence calls from 4 variant callers (via SMuRF). **b** Each genomic position selected for training is encoded as a multi-dimensional matrix of reads and associated features (e.g. base quality and mapping quality) and fed to a CNN for training. Source data are provided as a Source Data file.

VarNet was trained on data from over 300 matched normal and tumor genomes comprising seven cancer types (lung, sarcoma, colorectal, lymphoma, thyroid, liver, and gastric cancers). All samples were whole-genome sequenced (WGS) at depths of 50–150×. Since ground-truth labels were unavailable, an ensemble method (SMuRF) was used to generate mutation calls (SNV and indels) from callsets of four popular mutation callers in the bcbio-nextgen pipeline (see the “Methods” section). Training datasets containing equal numbers of mutated and non-mutated sites were created to train two deep learning models, one for SNV calling (2.5M sites) and the other for indel calling (2.1M sites) (Fig. 1a). Image-like representations of these sites are generated using the information in raw alignments overlapping these sites such as base, base quality, mapping quality, strand bias as well as the reference base. These properties are numerically encoded at each candidate site in distinct input channels along with the surrounding sequence context of neighboring sites so the model can learn relevant mutational signatures of alignment properties. Deep convolutional networks were then trained on these image-like representations to predict the probability of mutation at each site.

### Benchmarking on real tumor samples

**Fig. 2:**
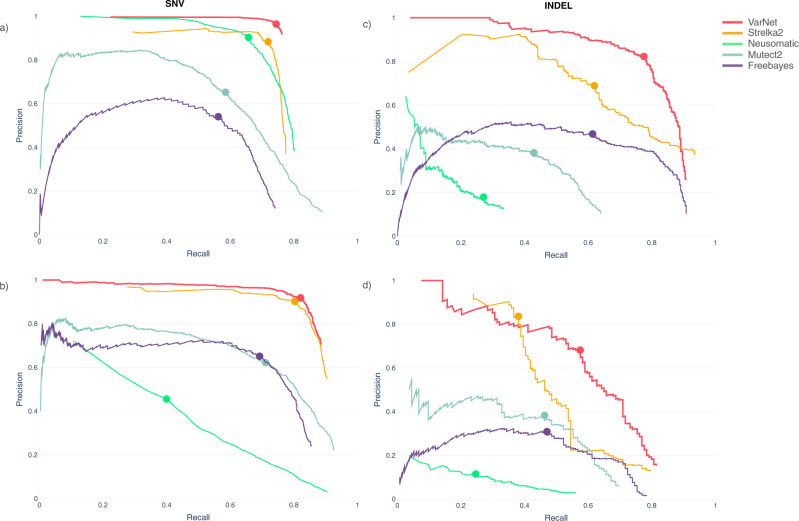
Variant calling accuracy on ICGC tumors. **a**, **b** Precision/recall curves for SNV calling in the MBL and CLL samples, respectively. **c**, **d** Precision/recall curves for indel calling in the MBL and CLL samples, respectively. Solid circles indicate the highest *F*1-accuracy score obtained for each method. Source data are provided as a Source Data file.

We tested the performance of VarNet on independent and publicly available benchmark datasets comprising both real and in-silico generated mutations. The International Cancer Genome Consortium (ICGC) Gold Set comprises verified somatic mutations in chronic lymphocytic leukemia (CLL) and medulloblastoma (MBL) tumor-normal pairs that were identified using high-coverage (~300×) whole-genome sequencing (WGS) data from multiple sequencing centers and further curated through manual review^7^. We downsampled the original high-coverage (~300×) tumor–normal WGS data to coverage levels commonly adopted for tumor WGS (~100×). We then evaluated the generalization performance of VarNet for both SNV and indel calling on these two samples. Overall, VarNet made calls at higher precision and recall compared to other callers for both SNVs and indels (Fig. 2). On the MBL sample, VarNet outperformed other callers achieving accuracy (*F*1) scores of 0.84 (SNV) and 0.79 (indel), compared to Strelka2’s 0.79 (SNV) and 0.65 (indel), and Mutect2’s 0.68 (SNV) and 0.40 (indel). On CLL, VarNet again outperformed other callers achieving *F*1 scores of 0.87 (SNV) and 0.62 (indel) whereas Strelka2 achieved 0.85 (SNV) and 0.52 (indel). NeuSomatic, which was trained on mutations from a synthetic tumor sample, performed inconsistently on these real tumor samples, achieving *F*1 scores of 0.43 (CLL) and 0.76 (MBL) for SNV calling, and 0.16 (CLL) and 0.22 (MBL) for indel calling.

**Fig. 3:**
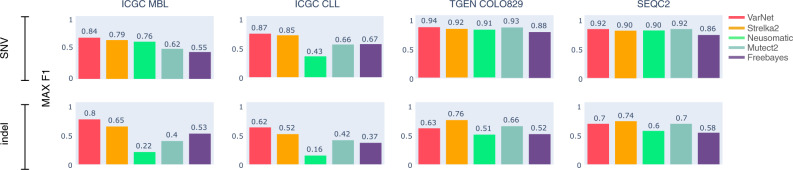
Variant calling accuracy across real tumor datasets. Maximum *F*1-accuracy scores achieved by methods on real tumor benchmark samples (MBL: medulloblastoma, CLL: chronic lymphocytic leukemia, COLO829: melanoma, SEQC2: breast cancer) for SNV calling (top) and indel calling (bottom). Source data are provided as a Source Data file.

We further benchmarked callers on COLO829, a metastatic melanoma cell line with a multi-institutionally defined reference set of somatic mutations^8^, and a SEQC2 established somatic reference callset derived from a breast cancer cell line^9^. These two somatic reference callsets were created by a consensus approach using data from multiple sequencing and variant calling pipelines. The SEQC2 reference callset was partially validated using targeted sequencing (>2000-fold coverage) to establish high confidence calls. For SNV calling on COLO829, all callers performed well with VarNet achieving the highest *F*1-score (0.94) (Fig. 3 and Supplementary Fig. 6a). For indel calling, Strelka2 and Mutect2 achieved higher accuracy (0.76 and 0.66) than VarNet (0.63) (Fig. 3 and Supplementary Fig. 6b). For SNV calling on the SEQC2 reference callset, VarNet achieved the highest *F*1-score (0.92) along with Mutect2 (0.92) (Fig. 3 and Supplementary Fig. 7). Strelka2 achieved the highest *F*1-score (0.74) for indel calling followed by VarNet (0.70) and Mutect2 (0.70).

In summary, on SNV calling in real tumor samples, VarNet outperformed all other callers in our analysis (avg. max. *F*1-score = 0.89), ahead of current methods such as Strelka2 (0.85) and Mutect2 (0.74). On indel calling, VarNet also often outperformed existing callers with an average *F*1-score of 0.69, followed by Strelka2 (0.64) and Mutect2 (0.49). Overall, VarNet showed accurate and consistent performance when evaluated on real tumor benchmarks.

### Performance on low variant allele fraction mutations

**Fig. 4:**
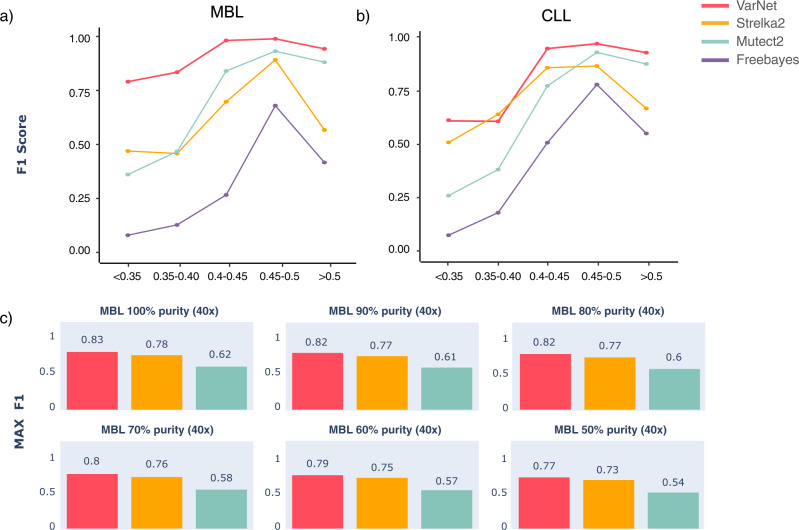
Impact of VAF and tumor purity levels. **a**, **b** SNV calling at VAF level bins in the MBL/CLL tumors. **c** SNV calling on MBL downsampled to 40×-depth and “contaminated” with normal reads to simulate purity levels. Purity levels are relative to the original purity of the MBL tumor (>95%). Source data are provided as a Source Data file.

We next evaluated the impact of variant allele frequency (VAF) levels on VarNet’s accuracy. The MBL tumor sample consists of a tetraploid background combined with altered ploidy at five chromosomes while the CLL tumor sample comprises large copy number variants^7^. This genomic background generated somatic mutations at distinct VAF levels, enabling us to evaluate the performance of VarNet at different VAF ranges (Fig. 4a, b). For mutations with VAF < 0.3, VarNet had higher accuracy (average *F*1 score across CLL and MBL of 0.70) compared to Strelka2 (0.49), Mutect2 (0.31), and Freebayes (0.08). The performance of all methods expectedly improved at higher allele fractions. However, curiously, Strelka2 and Freebayes showed noticeably lower *F*1 scores at allele fractions >0.5 (29% and 35% lower than 0.45–0.5 range, respectively), potentially mistaking some somatic mutations to be germline mutations at higher VAFs. Overall, VarNet demonstrated high accuracy across both low and high VAF levels as compared to other callers.

### Performance at low tumor purity and read depth

We next evaluated the impact of tumor purity levels (fraction of cancer cells in tumor) as well as low read depths. The ICGC MBL sample was estimated to have high tumor purity (>95%) and an average read depth of ~300×^7^. Hence, we diluted the MBL sample with reads from the matched normal sample to simulate increasing levels of tumor impurity, as well as downsampled the tumor sample to 40× coverage to simulate low read depth (see the “Methods” section). The lowered read depth alone only had a minor effect on performance for all methods (~1%, Fig. 4c). All methods expectedly demonstrated lower recall with increasing levels of tumor impurity (Fig. 4c). At 70% purity (30% normal dilution), VarNet achieved the highest *F*1 score (0.80) ahead of Strelka2 (0.76) and Mutect2 (0.58). While VarNet’s recall dropped 7% from the original read depth (~100×) and purity, precision increased from 0.96 to 0.97, suggesting reliable calls even at low read depths and purity levels (Supplementary Fig. 8). At 50% tumor purity, VarNet still achieved the highest *F*1 score (0.77) ahead of Strelka2 (0.73) and Mutect2 (0.54). VarNet provided a recall of 0.64 at this lower purity level without any drop in precision (0.97).

### Benchmarking on DREAM challenge synthetic tumor samples

We further benchmarked VarNet on synthetic tumors from the DREAM Somatic Mutation Challenge^10^. This dataset comprises synthetic tumors generated from a cell line sequenced to 80× (split into normal and tumor samples) and where in silico generated SNVs and indels have been added to the tumor sample. On SNV calling, VarNet outperformed most other methods (Supplementary Figs. 3 and 4). VarNet achieved a top *F*1 score of 0.90 on average across the synthetic tumors, ahead of Strelka2 (0.86) and Mutect2 (0.81). The only method with better overall performance on the synthetic tumors was NeuSomatic, likely because this method was trained on a subset of the DREAM tumor data^4^. Indeed, while NeuSomatic had the highest SNV calling performance (*F*1 = 0.96) across the DREAM synthetic tumors, its performance was not consistent when tested on the ICGC real tumor samples (average *F*1 = 0.60, Fig. 2). On indel calling, VarNet achieved an average F1 score of 0.66 across DREAM tumors behind Strelka2 (0.68) and Mutect2 (0.81) (Supplementary Fig. 5). While Mutect2 achieved the best performance for indel calling in the DREAM synthetic tumors, it showed considerably lower performance on the real ICGC tumors (0.41), suggesting a high-variance model. In contrast, VarNet’s indel calling performance was consistent across real and synthetic tumors (*F*1 = 0.69 versus 0.66). Strikingly, the indel calling performance of Neusomatic was the lowest of all methods (average *F*1 = 0.09) across the DREAM synthetic tumors. Overall, VarNet performed consistently across real and synthetic tumors, often outperforming existing methods.

### Interpreting features exploited by VarNet

**Fig. 5:**
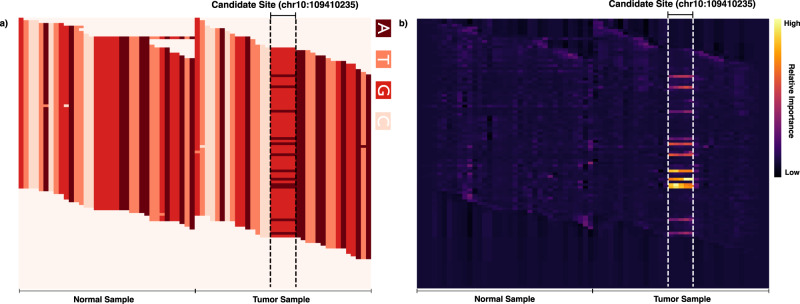
Inference of model activation. **a** VarNet encoding (base channel) of an SNV on chromosome 10 in MBL. The candidate position is repeated 5× in both the normal and tumor image. Variant alleles are visible at the candidate site in the tumor sample image. **b** Heatmap visualization showing ‘pixels’ in the base channel most important to VarNet’s deep learning model. VarNet has identified variant alleles at the candidate site in the tumor. Pixel-wise importance scores were computed using Guided Backpropagation11.

**Fig. 6:**
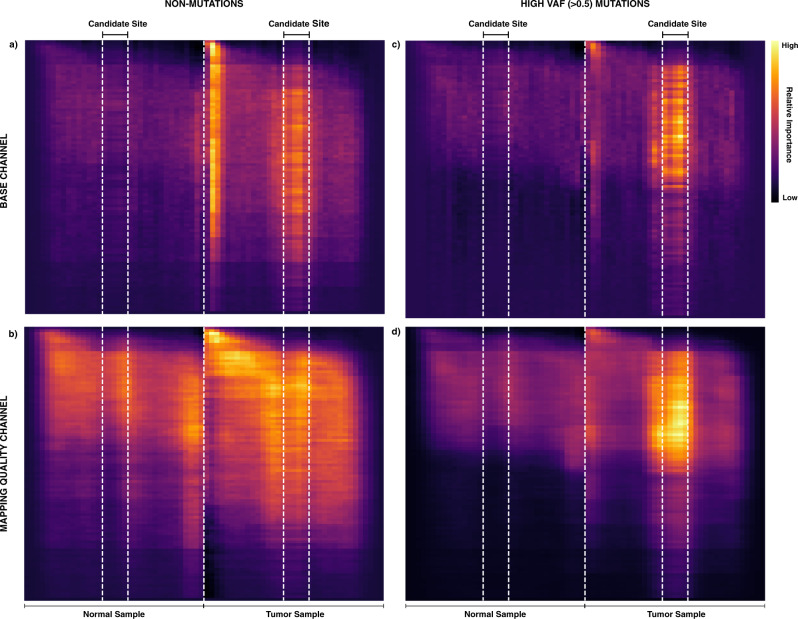
Model activation at mutated and non-mutated sites. Pixel importance scores are computed for individual sites using Guided Backpropagation^11^. Importance scores were averaged across 1000 randomly selected sites from the training set. **a**, **b** Heatmap of averaged pixel importance at non-mutated sites for the base and mapping quality channel, respectively. **c**, **d** Heatmap of averaged pixel importance at High-VAF-mutated sites for the base and mapping quality channel, respectively. Visualizations for other input feature channels are provided in Supplementary Fig. 9.

Finally, we sought to interpret the features learned by VarNet’s deep learning model. To aid interpretation, we generated heatmaps of importance assigned by VarNet to individual pixels in its input using guided backpropagation, which is a technique that uses model gradients to assign importance scores to pixels^11^. We visualized these pixel importance scores using heatmaps to illustrate VarNet’s ability to identify variant alleles at an individual mutated site (Fig. 5) as well as an average across many randomly selected sites to interpret commonly used features (Fig. 6). Although VarNet’s deep learning model is not trained with any specialized knowledge of mutations or genomic data, these visualizations revealed how the model has learned to identify variant alleles at the candidate site in the tumor. High importance was assigned to pixels containing individual variant alleles at the candidate site across all input channels including base and mapping quality. Activation for the mapping quality feature was evenly distributed across the input image for non-mutated positions (Fig. 6b) but showed higher importance at the mutated site and upstream bases in the presence of mutations (Fig. 6d). Positions upstream of the candidate mutation site are activated across input channels, potentially suggesting the use of the immediate sequence and read context by the model. The reference base channel showed higher activation for non-candidate sites suggesting it may not be important for predicting mutations in general and we observed no noticeable differences in pixel activation between low and high VAF mutations (Supplementary Fig. 9). All input channels indicated the highest activation at the tumor candidate site across both mutated and non-mutated inputs (Supplementary Fig. 9). Overall, these data demonstrate how VarNet uses multiple positions and properties of the encoded alignment images to predict mutations.

## Discussion

We have described an accurate deep learning approach for somatic variant calling using matched tumor and normal sequencing data. Compared to existing callers, VarNet takes a unique approach that does not use human-engineered features to predict mutations. Instead, we train the deep learning models on rich representations of raw sequence alignments. Conceptually, this process is mimicking how human experts often manually visualize and curate somatic mutations.

In contrast to Neusomatic^4^, an existing deep learning-based somatic mutation caller, VarNet was trained on real mutations in multiple cancers and exploits raw alignment data. While Neusomatic summarizes feature statistics of all alignments in a 7 bp window around candidate sites, VarNet encodes raw alignments in a larger (30 bp for SNV; 70 bp for indels) sequence context to learn signatures of somatic mutations. We further demonstrated the effectiveness of our input encoding and training strategy by directly benchmarking VarNet and NeuSomatic using the same training cohort (Supplementary Note 1).

We performed genome-wide benchmarking of VarNet on different real tumors and demonstrated best-in-class accuracy and ability to generalize to simulated mutations as well. Moreover, VarNet was able to achieve robust performance in challenging regions of the genome that are not highly *alignable*^12^ (Supplementary Note 2). Intriguingly, VarNet was also able to outperform the ensemble calling method used to generate pseudo-labels in the training dataset, on independent benchmarks (Supplementary Figs. 3–5). These results suggest that the deep learning approach is able to successfully learn and generalize when presented with sufficiently large datasets using weak supervision.

Yet, there still remain significant challenges for somatic variant calling. As our independent benchmarks have demonstrated, indel calling remains a significant challenge for all existing callers. Notably, we found in our experiments that indel calling benefited from additional training samples more than SNV calling, since indels occur at lower rates than point mutations and are also more difficult to pseudo-label accurately (see the “Methods” section). As additional tumor datasets become available, VarNet could leverage more training data through weak supervision or potentially pseudo-labels generated by VarNet itself (self-training) to improve indel calling performance. This self-training technique has been successfully exploited by other deep learning approaches for biological problems, e.g. AlphaFold2^13^.

In conclusion, we present an accurate method for somatic variant calling, demonstrating how a scalable deep learning approach could augment and potentially supplant human-engineered features and heuristic filters in somatic variant calling.

## Methods

### Training data

Training data was generated using WGS tumor data from regional hospitals and research institutes including National University Hospital Singapore, National Cancer Centre Singapore, Genome Institute of Singapore as well as TCGA (https://www.cancer.gov/tcga). All samples in training cohorts were obtained with written informed consent from patients. Analysis of local samples was approved by the institutional review boards at National Cancer Center Singapore and National University Hospital Singapore. The data repository included 356 matched tumor/normal samples across seven cancer types i.e., lung, thyroid, colorectal, sarcoma, gastric, liver, and lymphoma (see Supplementary Table 6). The gastric^14^ and liver^15^ cancer cohorts were obtained from previously published studies after signing data-use agreements. Samples were sequenced using Illumina HiSeq (Paired-End, medium depth 50–150×) and processed by the bcbio-nextgen^16^ pipeline. Reads were aligned to GRCh37 using BWA-MEM^17^ followed by marking and removal of duplicate reads. GATK3^18^ with local realignment around indels was used for post-processing.

We generated pseudo-labels using SMuRF, an ensemble somatic mutation caller^3^. Ensembling multiple (noisy) labels is a theoretically justified^19^ and practically useful approach for weakly supervised learning^20^. The bcbio-nextgen framework was used to generate somatic variant calls using four callers: MuTect2^1^, Freebayes somatic^21^, VarDict^22^, and VarScan^23^. Variant and auxiliary features generated by these callers were fed to SMuRF, which makes predictions using its random forest classifier.

A total of 2.5 million and 2.1 million training data points were generated for SNV and indel model training, respectively. Fewer indel sites were used for training as indels are generally less common in tumors than point mutations. Both SNV and indel training sets were class-balanced to contain equal numbers of mutated and non-mutated sites (non-mutated sites significantly outnumber mutated sites in any tumor). Calls made by SMuRF were chosen as mutated sites while sites that were not called by SMuRF but called by at least one of the four callers, were chosen as non-mutated sites. We reasoned that this approach would make the classification task more challenging and yield more discriminative information when training VarNet.

### Cancer-type bias and training set size

We tried to balance the trade-off between downsampling over-represented cancer types (e.g. colorectal cancer) to reduce bias and maintain the overall size of the training set. While down-sampling over-represented cancer types in the SNV training set improved the generalization performance of the SNV model, the indel model benefited from all available training data without balancing cancer types as indels are typically far less frequent than SNVs. Moreover, pseudo-labels for indels are expected to contain more errors than SNVs since variant callers are typically less accurate for indels. Larger training sets can alleviate this label noise when training machine learning models.

### Input encoding

For each candidate mutation site, aligned reads are encoded in an image-like representation with features including base, mapping quality, base quality, and strand bias; the reference base is encoded in a separate channel. Each base is assigned a distinct numerical value; deleted bases are also encoded by a unique numerical value different from that of A/T/G/C. Insertions are encoded in-place with adjustments to the reference base channel since inserted bases do not have corresponding loci in the reference. As most short-indels are <10 bp, indels no longer than 35 bp are encoded to fit within the image. Normal and tumor images are encoded adjacently (Fig. 1b). For each site, an input tensor (SNV: (100,70,5), indel: (140,150,5)) encodes the candidate as well as the surrounding sequence context in both tumor and normal so the model is able to learn relevant mutational signatures (Fig. 5a). The candidate site is repeated 5× in the SNV input-encoding to amplify the signal at the candidate mutation site. This is not done for indels as it would affect input-encoding width due to their variable length. The SNV model uses up to 100 overlapping alignments while the indel model can use up to 140. If read coverage exceeds this, alignments are randomly sampled.

### Deep-learning model and training

After experimentation with multiple architecture designs, we designed a convolutional neural network (ConvNet) for SNV calling while the InceptionV3 architecture was used for indel calling. The SNV calling model is composed of a convolutional neural network with 10 convolutional blocks each containing convolution, ReLu activation, and Batch Normalization^24^ layers. Two average-pooling layers are used to downsample information between blocks. Convolutional layers are followed by three densely connected (comprising 256, 128, and 64 units) layers that are followed by a sigmoid output layer that computes the probability of mutation. There are ~3.5 million trainable parameters in the SNV model. For indel calling, a larger model, Inceptionv3^25^, was used. Both models were trained with the Adam^26^ optimizer with an initial learning rate of 1e−4 and a mini-batch size of 32. Tensorflow^27^ was used to train models on a Nvidia Titan-X GPU.

### Genome pre-filtering

VarNet takes as input binary alignment map (BAM) files of matched tumor–normal pairs. For new samples, as most sites in the sample are unlikely to be mutated (e.g. contain no variant alleles), VarNet first filters positions that have a very low likelihood of being a somatic mutation (Supplementary Tables 1 and 2). The goal of this pre-filtering is to reduce computation cost while retaining high sensitivity for mutated sites (Supplementary Tables 3 and 4). After filtering, candidate sites are processed using the trained deep learning models. VarNet also accepts browser extensible data (BED) files of genomic regions to restrict mutation calling and filtering, which is useful for Exome sequencing data.

### Germline variant filtering

VarNet performs germline variant filtering, without local re-assembly and haplotype determination, of its somatic variant callset to remove calls with high likelihood of being germline variants, i.e., variant alleles with significant (>10%) representation in the normal sample. VarNet scans the neighboring 10 bp window around each site in its somatic callset to identify suspected germline SNPs and short-indels. VarNet then filters those somatic mutations that overlap or are within one base pair of an identified germline SNP or indel. This filtering procedure is performed as post-processing of the somatic callset, hence, its computational cost is minimal with sensitivity comparable to that of using a standalone germline variant caller.

### Test datasets and in silico dilutions

Benchmark datasets, MBL and CLL^7^, COLO829^8^, DREAM tumors^10^ were processed similarly to the training data. The SEQC2 reference sample^9^ was aligned to GRCh38 and processed using GATK4. MBL and CLL samples were downsampled from their original high read depths (~300×) to commonly used whole-genome sequencing read depths (~100×). For benchmarking callers on the SEQC2 reference sample, we used a Illumina HiSeq sample (WGS_IL_N_1,WGS_IL_T_1; https://sites.google.com/view/seqc2/home/sequencing) and restricted our evaluation to the high confidence regions established by the consortium. The MBL tumor and normal samples were further subsampled and merged using SAMtools^28^ at various proportions to simulate distinct tumor purity levels.

### Performance metrics

We have reported precision-recall curves for all benchmarks, where *precision* (*positive predictive value*) refers to the percentage of predicted mutations that are correct, and *recall* refers to the percentage of true mutations that were correctly identified. We varied scores produced by each caller (VarNet’s Score, Strelka2’s SomaticEVS, Mutect2’s TLOD, Freebayes’ ODDS, NeuSomatic’s Score, Varscan’s SSC) to generate precision-recall curves for each method. We have also reported *F*1 scores, which is the harmonic mean of precision and recall.

### Reporting summary

Further information on research design is available in the Nature Research Reporting Summary linked to this article.

## Supplementary information

Supplementary Information

Reporting Summary

## Data Availability

Sequence data for all benchmark samples are from previously published studies. The MBL data are available in the European Genome-Phenome Archive (EGA) under accession code EGAD00001001859, the CLL data are available in EGA under accession code EGAD00001001858, the COLO829 data are available in EGA under accession code EGAD00001002142, the SEQC2 data are available in the Sequence Read Archive (SRA) under accession codes SRX4728512 and SRX4728509. The first three ICGC-TCGA DREAM Somatic Mutation Calling Challenge synthetic samples are available from SRA under accession codes SRX570726, SRX1025978, and SRX1026041. The remaining synthetic samples, i.e., DREAM4 and DREAM5, are available upon request through the ICGC Data Access Compliance Office (https://daco.icgc-argo.org/). Sequence data for the gastric cancer training cohort are from a previously published study and available from EGA under accession code EGAD00001000782, the liver cancer training cohort is also from a previously published study and available upon request through Genomic Data Commons (GDC) [https://portal.gdc.cancer.gov/projects/TCGA-LIHC] (instructions to obtain access can be found here: https://gdc.cancer.gov/access-data/obtaining-access-controlled-data). Sequence data for the remaining training cohorts, i.e., sarcoma, lymphoma, colorectal, thyroid, and lung, are available upon request due to a lack of patient consent to deposit in a repository. Requests for access will be processed within 1 month subject to the signing of a data-use agreement (e-mail skanderupamj@gis.a-star.edu.sg), access will be provided for the duration of the project requiring the data. Source code is described in the “Code availability” section. Source data for figures and tables are provided with this paper. Source data are provided with this paper.

## Source data

Source Data file

## Code availability

VarNet is available as a Python package at https://github.com/skandlab/VarNet. The results in this paper were based on VarNet v1.1.0^29^. VarNet relies on NumPy^30^, Tensorflow^31^, Pysam (https://github.com/pysam-developers/pysam), pandas^32,33^, Pybedtools^34,35^, and Joblib (https://github.com/joblib/joblib). A Docker image containing all required libraries is also available, described in the repository. Computational requirements are reported in Supplementary Table 5.
