# Supplementary material for: Accurate somatic variant detection using weakly supervised deep learning
Source: Nat Commun. 2022 Jul 22;13:4248. doi: 10.1038/s41467-022-31765-8 (PMC9307817; doi:10.1038/s41467-022-31765-8)
Supplement: Supplementary file 1 — Supplementary Information [file 41467_2022_31765_MOESM1_ESM.pdf]

Accurate somatic variant detection using weakly supervised deep learning

Supplementary Information

Supplementary Note 1	2
Supplementary Note 2	5
Supplementry Figure 3	6
Supplementry Figure 4	7
Supplementry Figure 5	8
Supplementry Figure 6	9
Supplementry Figure 7	10
Supplementry Figure 8	11
Supplementry Figure 9	12
Supplementary Table 1	13
Supplementary Table 2	14
Supplementary Table 3	15
Supplementary Table 4	16
Supplementary Table 5	17
Supplementary Table 6	18

Supplementary Note 1

Benchmarking VarNet and NeuSomatic trained on the same cohort

VarNet and NeuSomatic are deep-learning based callers with key differences in their modeling and training strategies. In order to compare the modeling and training approaches, we benchmarked the callers after training them on the same cohort of samples. Using a matched training cohort provided a controlled setting to compare their different approaches. We created a training set comprising 100K mutations (SNVs), similar to the training set used in NeuSomatic, derived from matched normal-tumor samples comprising gastric, colorectal and lymphoma cancers. We trained NeuSomatic using two strategies, a.) In silico mutation spike-in (following NeuSomatic's recommendation) and b.) training on real mutations using pseudo-labels generated by SMuRF (as proposed in VarNet), to evaluate the effectiveness of the training strategies. We also trained VarNet from scratch on this cohort using weak supervision. We briefly describe the models that were trained:

NeuSomatic-100K-in-silico:

We spiked in mutations in normal replicates in the training cohort designated as the tumor. As we did not have access to high-coverage normal samples in the training cohort, we generated normal replicates by randomly subsampling (with replacement) each normal sample at 80% read-coverage, twice. We found this approach lead to better performance than using both matched normal and tumour samples in the cohort. We used the docker pipeline recommended in NeuSomatic to create in-silico tumours BAMSurgeon (https://github.com/bioinform/neusomatic#creating-training-data). We generated in-silico tumors comprising 100K mutations, in total. *Neusomatic-100K-in-silico* was trained on these in-silico mutations using NeuSomatic's training pipeline (https://github.com/bioinform/neusomatic) with default parameters.

***NeuSomatic-100K-real*:**

We trained another NeuSomatic model on real mutations using pseudo-labels generated by SMuRF. We used the same genomic regions used to train *NeuSomatic-100K-in-silico* as well as the same number of mutations. We trained *NeuSomatic-100K-real* from scratch on this dataset in the same fashion as *Neusomatic-100k-in-silico*.

***VarNet-100K-real*:**

We trained *VarNet-100K-real* from scratch on the same training data as *NeuSomatic-100K-real* to compare these methods when trained on the same mutations.

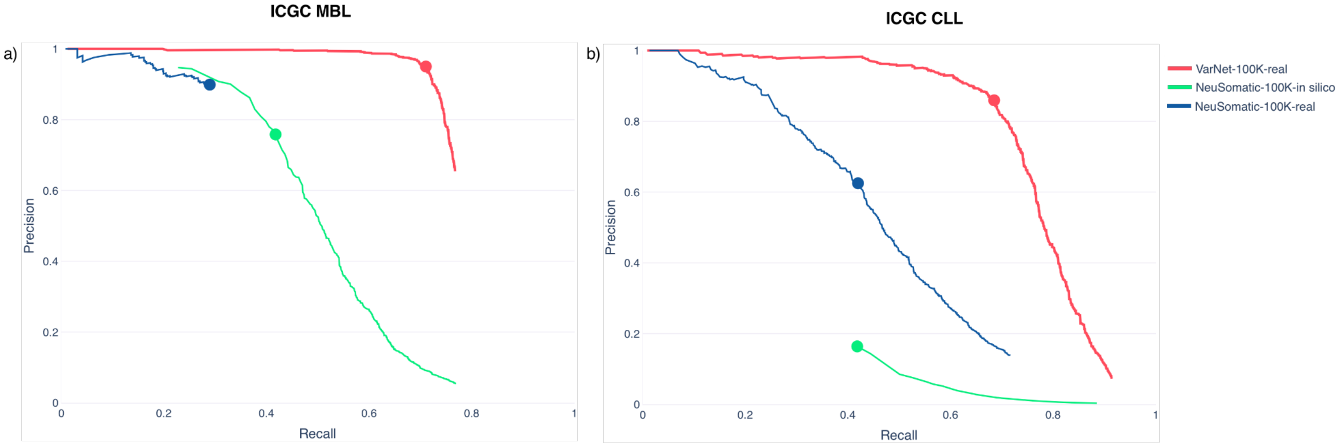

<u>Supplementary Fig. 1:</u> Benchmarking VarNet and NeuSomatic trained on the same cohort. Precision-recall (PR) curves for SNV calling on **a** MBL and **b** CLL. Source data are provided as a Source Data file.

<u>Results:</u> We evaluated these models on real tumor samples - ICGC MBL and ICGC CLL (Supplementary Fig. 1). Across both samples, VarNet-100K-real achieved significantly higher F1-scores (avg: **0.79**) than both NeuSomatic-100K-in-silico and NeuSomatic-100K-real. NeuSomatic-100K-in-silico achieved average F1-score **0.39** whereas NeuSomatic-100K-real achieved **0.47**. NeuSomatic-100K-real provided less sensitivity and overall F1 performance compared to VarNet-100K-real, which was trained on the same mutations and regions. It is worth noting that training NeuSomatic on real mutations improved over training on in-silico mutations. These results demonstrate the effectiveness of VarNet's model as well as the training methodology using real mutations and weak supervision.

Supplementary Note 2

Performance in low-alignability genomic regions

While aligners can confidently align reads to most regions of the genome, there are regions that are intrinsically difficult to align due to the presence of repetitive sequences. *Alignability* is a metric that is used to measure the confidence with which a genomic region can be aligned to. Since it is not possible to confidently align reads in low *alignability* regions even with high read-coverage or the absence of sequencing errors, callers that make fewer errors and calls in these challenging genomic regions are prefered. For this analysis, we used alignability data tracks from the ENCODE consortium to retrieve scores for the human reference genome (https://genome.ucsc.edu/cgi-bin/hgFileUi?db=hg19&g=wgEncodeMapability). We used the 75mer track for conservative alignability scores, although we obtained similar results using the 100mer track. We used all SNV PASS calls made by callers at default filter thresholds (VarNet's SCORE: 0.5, Strelka2's SomaticEVS: 7, Mutect2's TLOD: 6.3) to investigate their relative performance in regions of varying complexity. We averaged F1-scores for SNV-calling across three real tumor samples (CLL, MBL and TGEN-COLO829) in regions with different alignability scores. Compared to Strelka2 and Mutect2, VarNet achieved higher F1-scores in low-alignability regions and made most of its FP errors in high-alignability regions with few FPs in low-alignability regions (Supplementary Fig. 2). This suggests VarNet can effectively utliize read mapping-quality scores to make fewer errors in complex regions.

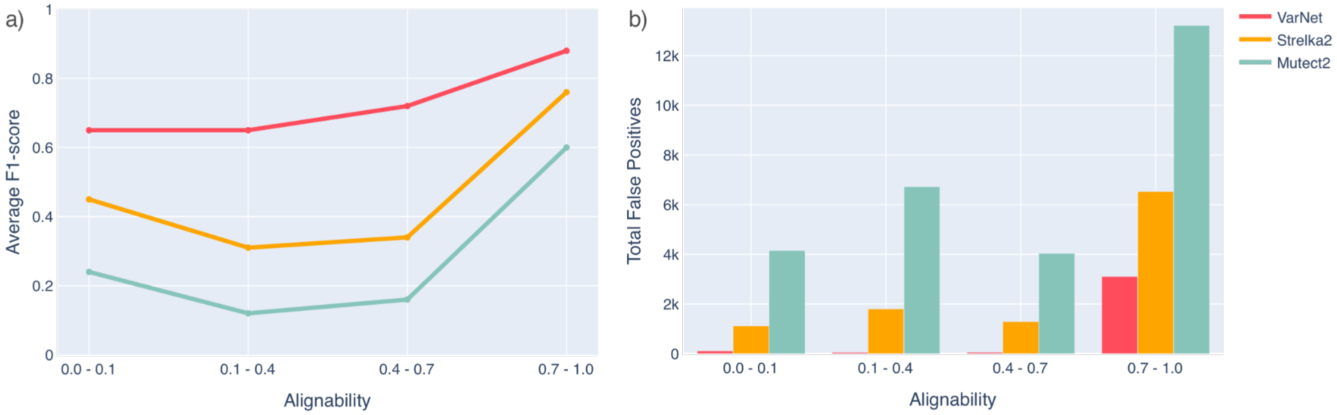

Supplementary Fig. 2: Performance in low-alignability regions. a F1-scores for SNV calling evaluated in genomic regions with different alignability scores, averaged across ICGC MBL, ICGC CLL and TGEN-COLO829. **b** Total number of false positive somatic SNV calls made by callers across ICGC MBL, ICGC CLL and TGEN-COLO829 in genomic regions with different alignability scores. Source data are provided as a Source Data file.

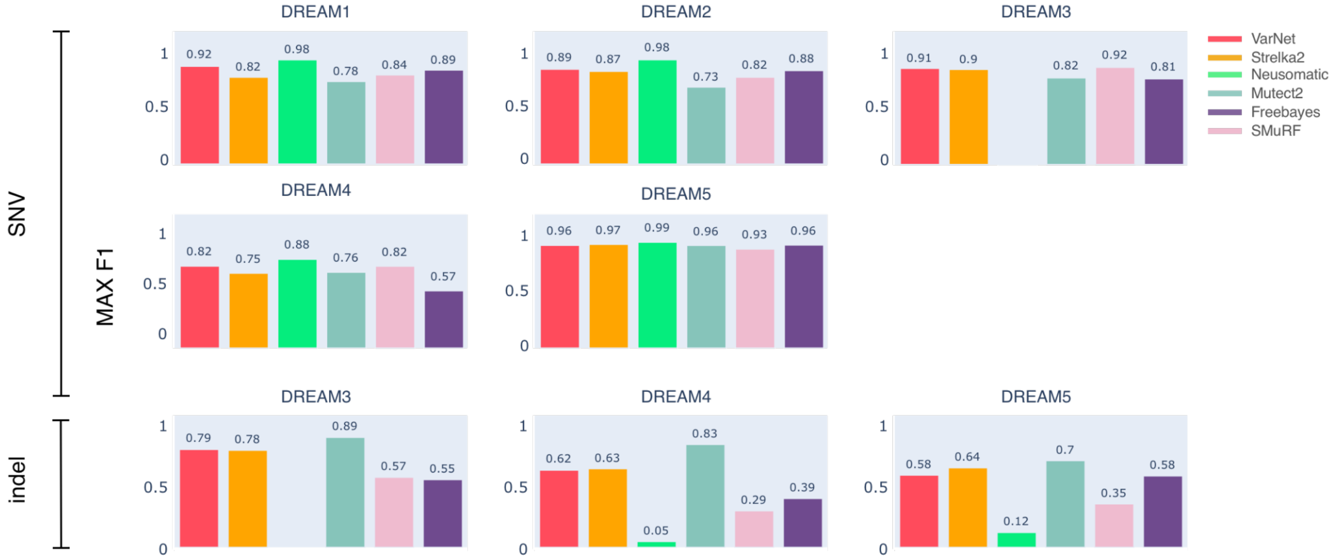

Supplementary Fig. 3: Max-F1 scores achieved by methods on synthetic tumors. Neusomatic was excluded from the DREAM3 benchmark as it was trained on that tumor dataset. Source data are provided as a Source Data file.

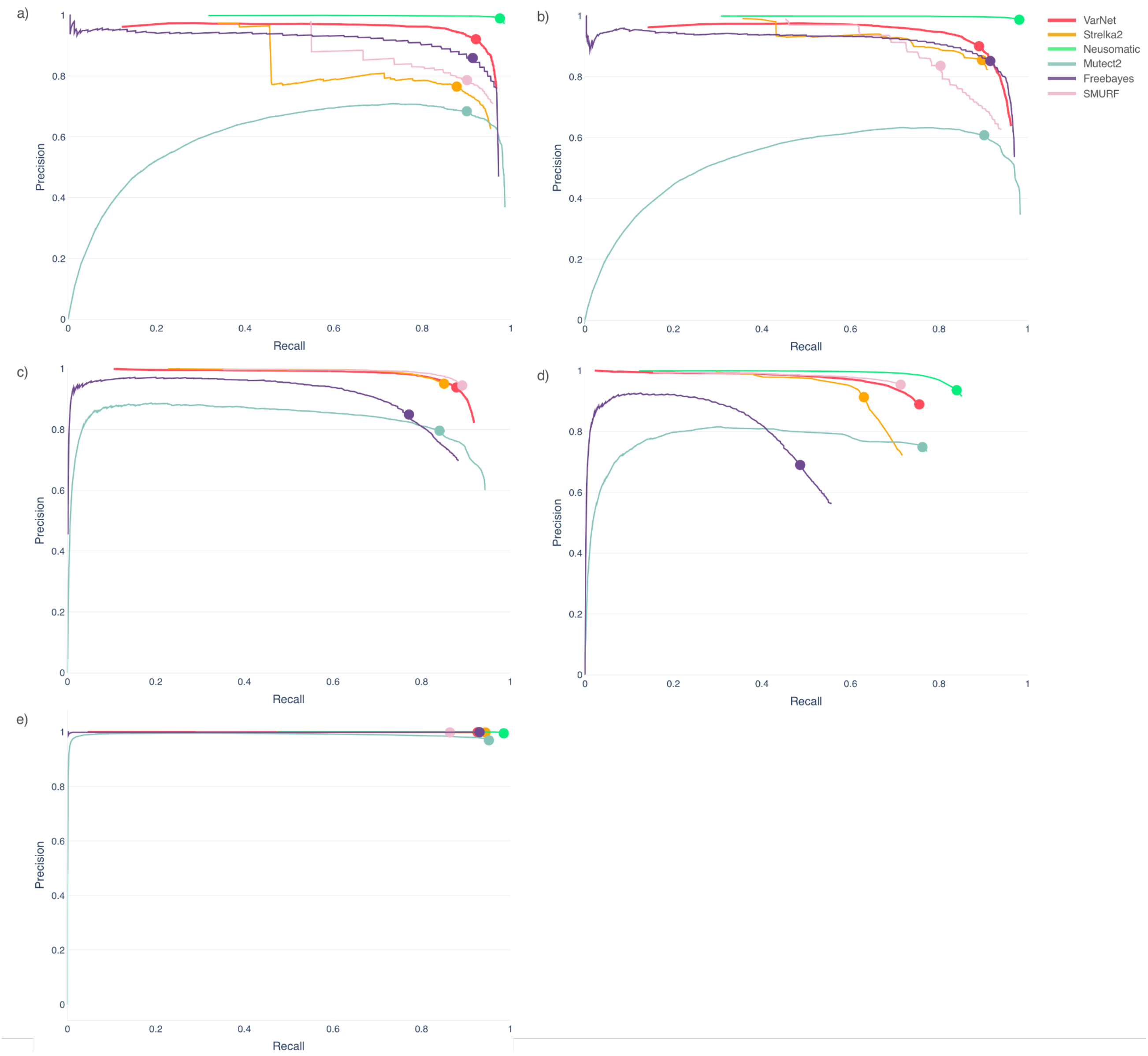

Supplementary Fig. 4: Precision-recall curves for SNV calling on DREAM tumors. **a** DREAM 1 **b** DREAM 2 **c** DREAM 3 **d** DREAM 4 **e** DREAM 5. Neusomatic was excluded from DREAM3 benchmark as it was trained on this tumor. Source data are provided as a Source Data file.

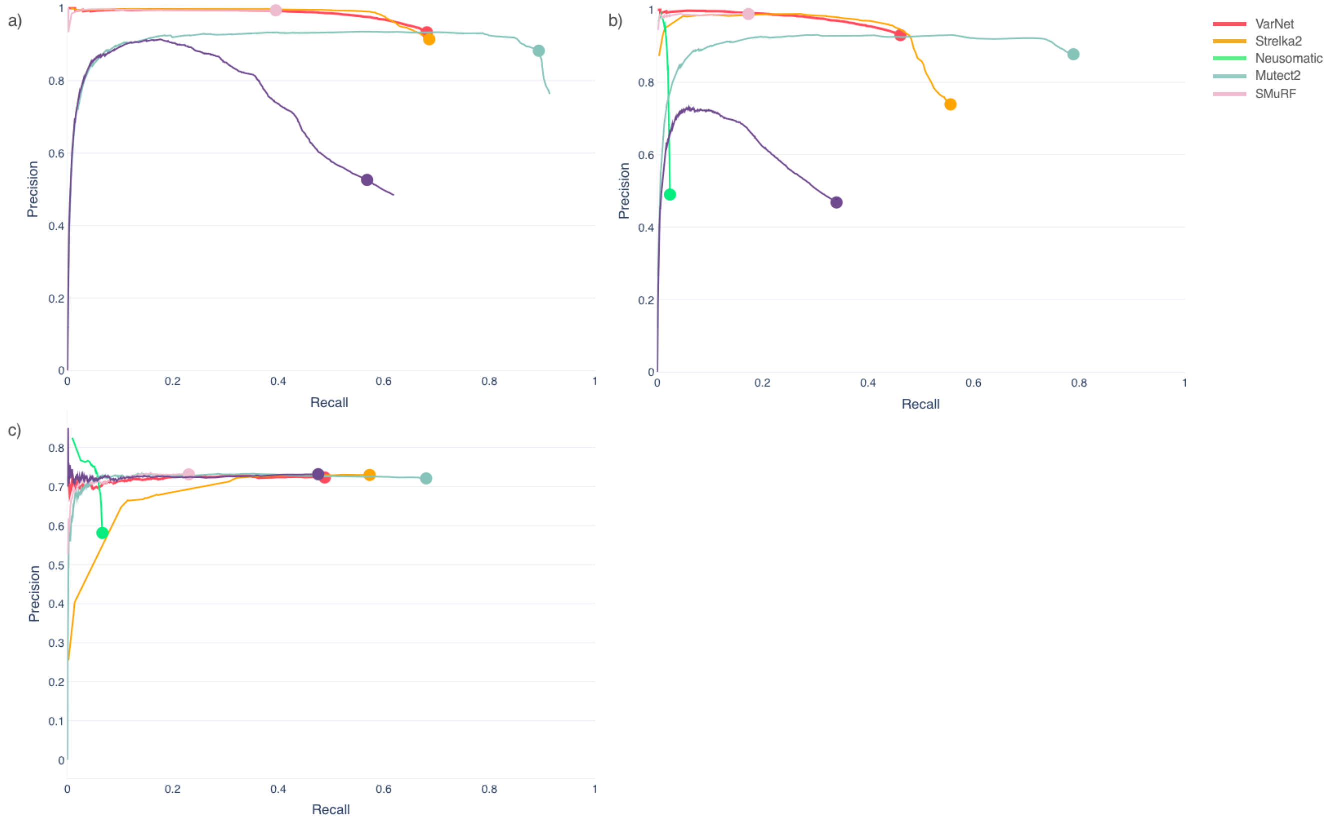

<u>Supplementary Fig. 5:</u> Precision-recall curves for indel calling on DREAM tumors. **a** DREAM 3 **b** DREAM 4 **c** DREAM 5. Neusomatic was excluded from DREAM3 benchmark as it was trained on this tumor. Source data are provided as a Source Data file.

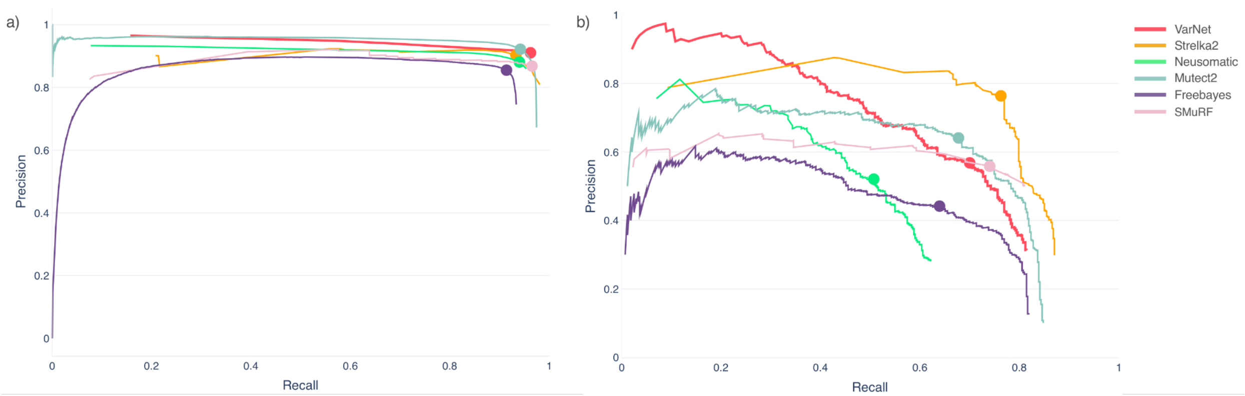

<u>Supplementary Fig. 6:</u> Benchmarking on TGEN-COLO829. a PR curves for SNV-calling. **b** PR curves for indel-calling. Source data are provided as a Source Data file.

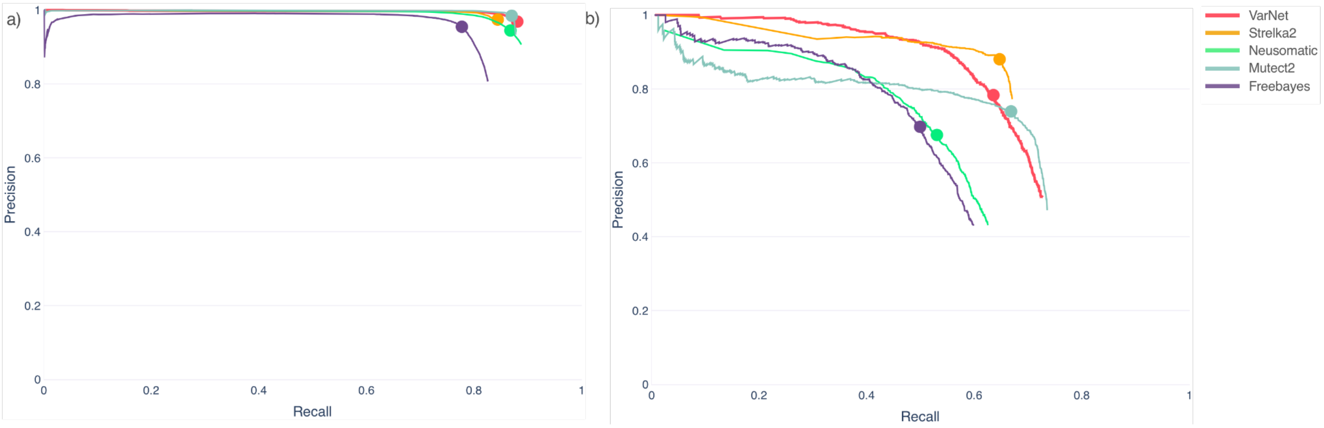

<u>Supplementary Fig. 7:</u> Benchmarking on the SEQC2 established benchmark sample. a PR curves for SNV-calling. **b** PR curves for indel-calling. Source data are provided as a Source Data file.

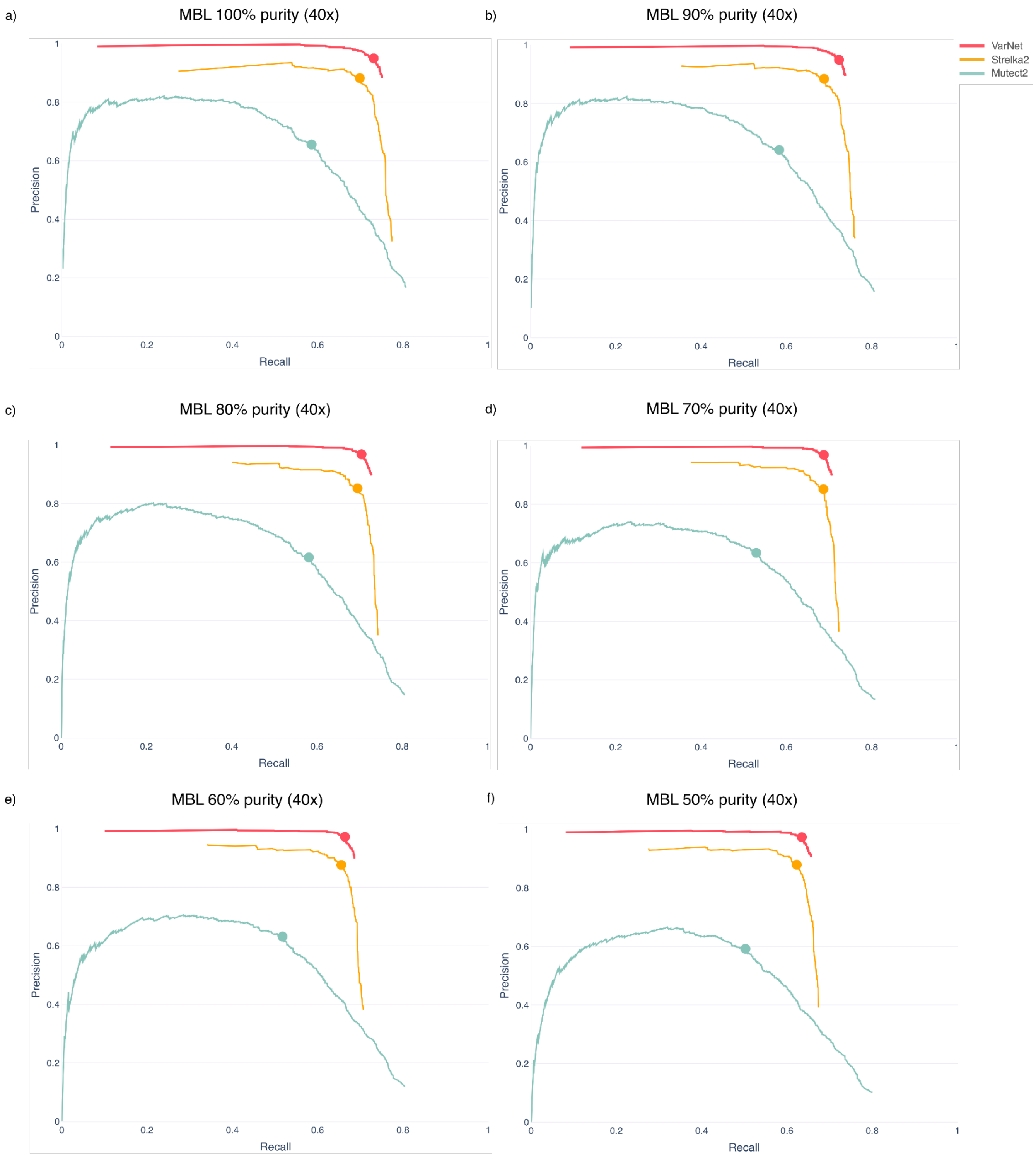

<u>Supplementary Fig. 8:</u> Performance at variable tumor purity levels. PR curves for SNV calling on MBL at variable tumor purity levels (downsampled to 40x). Purity levels were created by diluting with reads from the matched normal sample. 100% tumor purity refers to the original purity of the tumor sample. **a** 100% tumor purity. **b** 90% tumor purity. **c** 80% tumor purity. **d** 70% tumor purity. **e** 60% tumor purity. **f** 50% tumor purity. Source data are provided as a Source Data file.

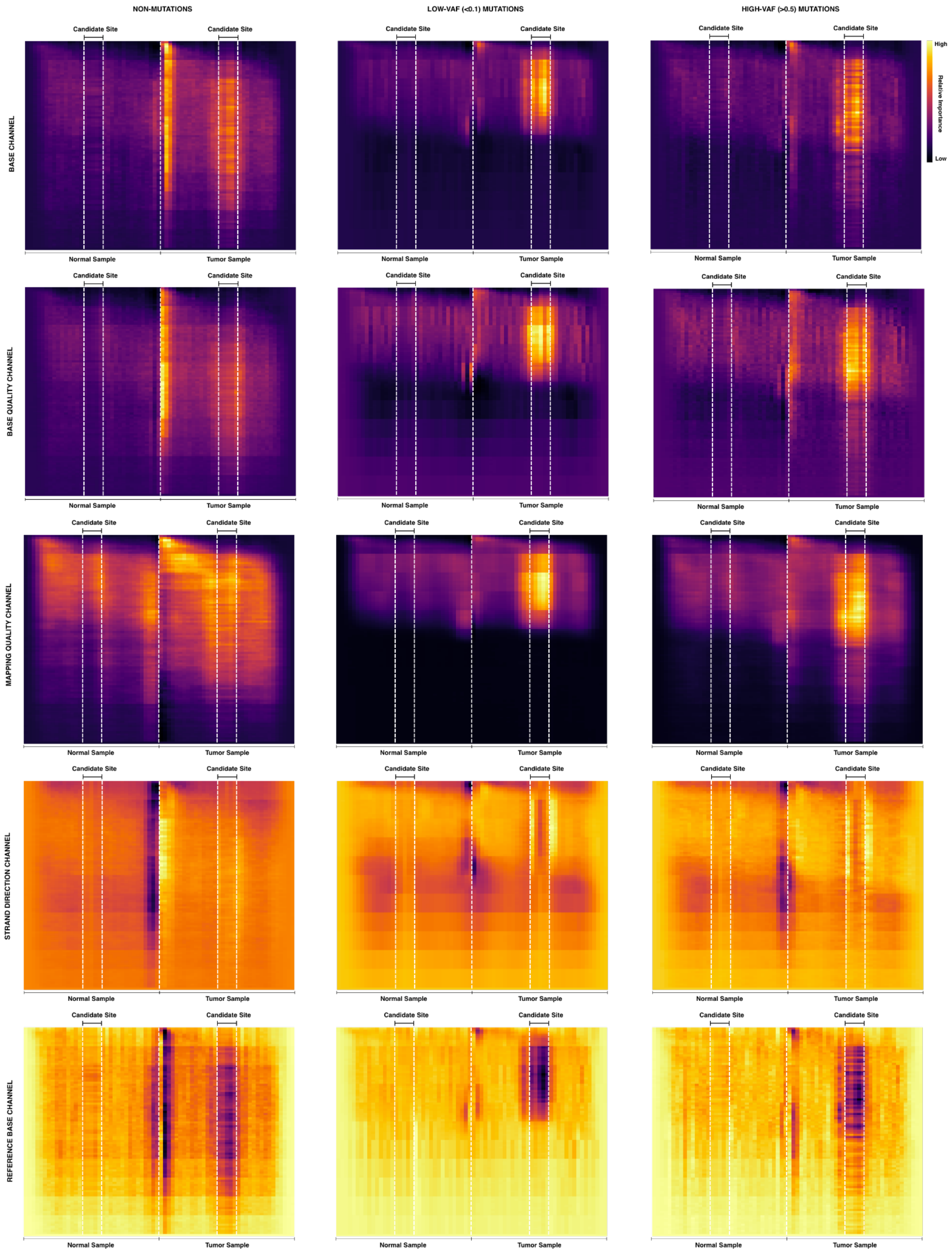

Supplementary Fig. 9: Model activation at mutated and non-mutated sites. A heatmap visualization of parts of the input the SNV model focuses on for predicting mutations (computing using Guided Backpropogation for a random sample of 200 mutations in the training set. The heatmap is averaged across the 200 samples and has been resized for compactness). As seen here, the model focuses most on the genomic position of interest in the tumor, among other positions in the input to predict mutations. The model has learned to identify the mutated site in the input without explicitly being specified during training.

FEATURE	THRESHOLD
MIN READ COVERAGE	7
MIN MUTANT ALLELE READS IN TUMOR	2
MIN READ MAPPING QUALITY	10
MAX MUTANT ALLELE FREQ IN NORMAL	0.05
MIN MUTANT ALLELE FREQ IN TUMOR	0.035
MIN BASE QUALITY	22

Supplementary Table 1: Filters applied during whole genome pre-filtering to identify SNV mutation candidates. These candidates are later processed by the deep learning model. Source data are provided as a Source Data file.

FEATURE	THRESHOLD
MIN READS WITH INDEL IN TUMOR	2
MIN READ MAPPING QUALITY FOR INSERTIONS	30
MIN READ MAPPING QUALITY FOR DELETIONS	20
MIN BASE QUALITY	22
MIN VARIANT ALLELE FRACTION	0.03

Supplementary Table 2: Filters applied during whole genome pre-filtering to identify indel mutation candidates.These candidates are later processed by the deep learning model. Source data are provided as a Source Data file.

SAMPLE	TRUE SNVS	SENSITIVITY
ICGC CLL	1,319	1,292 (0.980)
ICGC MBL	1,263	1,166 (0.923)
COLO829	35,542	35,191 (0.990)
SEQC2	39,447	38,350 (0.972)
DREAM1	3,537	3,494 (0.988)
DREAM2	4,332	4,301 (0.993)
DREAM3	7,903	7,720 (0.977)
DREAM4	16,315	15,753 (0.966)
DREAM5	45,383	45,250 (0.997)

Supplementary Table 3: Sensitivity of VarNet's whole genome SNV pre-filtering on benchmark samples. Pre-filtering identifies SNV candidates that are processed by the deep learning model. Source data are provided as a Source Data file.

SAMPLE	TRUE indels	SENSITIVITY
ICGC CLL	134	129 (0.963)
ICGC MBL	347	343 (0.988)
COLO829	446	430 (0.964)
SEQC2	1,625	1,575 (0.97)
DREAM3	7,991	7,037 (0.881)
DREAM4	14,230	11,856 (0.833)
DREAM5	16,428	10,839 (0.660)

Supplementary Table 4: Sensitivity of VarNet's whole genome indel pre-filtering on benchmark samples. Pre-filtering identifies indel candidates that are processed by the deep learning model. Source data are provided as a Source Data file.

PROCESSING	CPU HRS	MEMORY PER PROCESS
Genome Filtering	10-12	6GB
Prediction	150	10GB

<u>**Supplementary Table 5:**</u> **Expected whole genome run-time requirements for VarNet.** Wall-time requirements can be reduced by using multiple processes. Source data are provided as a Source Data file.

**Cancer Type**	**Cohort Type**	**# Samples**
Gastric	Public Cohort (EGA)	38
Liver	Public Cohort (GDC)	43
Sarcoma	Internal Training Cohort	23
Colorectal	Internal Training Cohort	164
Lymphoma	Internal Training Cohort	60
Thyroid	Internal Training Cohort	6
Lung	Internal Training Cohort	22

Supplementary Table 6: VarNet training cohorts and number of matched tumour samples. Source data are provided as a Source Data file.
