# Supplementary material for: Accurate somatic variant detection using weakly supervised deep learning
Source: Nat Commun. 2022 Jul 22;13:4248. doi: 10.1038/s41467-022-31765-8 (PMC9307817; doi:10.1038/s41467-022-31765-8)
Supplement: Supplementary file 2 — Reporting Summary [file 41467_2022_31765_MOESM2_ESM.pdf]

nature portfolio

Corresponding author(s): Anders Skanderup: skanderupamj@gis.a-star.edu.sg

Last updated by author(s): Jun 29, 2022

Reporting Summary

Nature Portfolio wishes to improve the reproducibility of the work that we publish. This form provides structure for consistency and transparency in reporting. For further information on Nature Portfolio policies, see our Editorial Policies and the Editorial Policy Checklist.

Statistics

For all statistical analyses, confirm that the following items are present in the figure legend, table legend, main text, or Methods section.

n/a	Confirmed	
☐	☒	The exact sample size (*n*) for each experimental group/condition, given as a discrete number and unit of measurement
☒	☐	A statement on whether measurements were taken from distinct samples or whether the same sample was measured repeatedly
☒	☐	The statistical test(s) used AND whether they are one- or two-sided<br>*Only common tests should be described solely by name; describe more complex techniques in the Methods section.*
☒	☐	A description of all covariates tested
☒	☐	A description of any assumptions or corrections, such as tests of normality and adjustment for multiple comparisons
☐	☒	A full description of the statistical parameters including central tendency (e.g. means) or other basic estimates (e.g. regression coefficient) AND variation (e.g. standard deviation) or associated estimates of uncertainty (e.g. confidence intervals)
☒	☐	For null hypothesis testing, the test statistic (e.g. *F*, *t*, *r*) with confidence intervals, effect sizes, degrees of freedom and *P* value noted<br>*Give P values as exact values whenever suitable.*
☒	☐	For Bayesian analysis, information on the choice of priors and Markov chain Monte Carlo settings
☒	☐	For hierarchical and complex designs, identification of the appropriate level for tests and full reporting of outcomes
☒	☐	Estimates of effect sizes (e.g. Cohen's *d*, Pearson's *r*), indicating how they were calculated

Our web collection on statistics for biologists contains articles on many of the points above.

Software and code

Policy information about availability of computer code

Data collection	bcbio-nextgen v1.0.7 (https://github.com/bcbio/bcbio-nextgen)<br>BWA-MEM v0.7.3 (https://github.com/lh3/bwa)<br>GATK v3.6 (https://github.com/broadinstitute/gatk-docs)<br>SAMTools v1.3
Data analysis	VarNet (v1.1.0) (https://github.com/skandlab/VarNet)<br>python3.7<br>Numpy v1.18.5<br>tensorflow v2.3.0<br>pysam v0.16.0.1<br>pandas v1.1.1<br>joblib v0.16.0<br>pybedtools v0.8.1

For manuscripts utilizing custom algorithms or software that are central to the research but not yet described in published literature, software must be made available to editors and reviewers. We strongly encourage code deposition in a community repository (e.g. GitHub). See the Nature Portfolio guidelines for submitting code & software for further information.

Data

Policy information about availability of data

All manuscripts must include a data availability statement. This statement should provide the following information, where applicable:

- Accession codes, unique identifiers, or web links for publicly available datasets
- A description of any restrictions on data availability
- For clinical datasets or third party data, please ensure that the statement adheres to our policy

Sequence data for all benchmark samples are from previously published studies. The MBL data are available in the European Genome-Phenome Archive (EGA) under accession code EGAD00001001859 [https://ega-archive.org/datasets/EGAD00001001859], the CLL data are available in EGA under accession code EGAD00001001858 [https://ega-archive.org/datasets/EGAD00001001858], the COLO829 data are available in EGA under accession code EGAD00001002142 [https://ega-archive.org/datasets/EGAD00001002142], the SEQC2 data are available in the Sequence Read Archive (SRA) under accession codes SRX4728512 and SRX4728509 [https://www.ncbi.nlm.nih.gov/sra/SRX4728512,https://www.ncbi.nlm.nih.gov/sra/SRX4728509]. The first three ICGC-TCGA DREAM Somatic Mutation Calling Challenge synthetic samples are available from SRA under accession codes SRX570726, SRX1025978 and SRX1026041 [https://www.ncbi.nlm.nih.gov/sra/SRX570726, https://www.ncbi.nlm.nih.gov/sra/SRX1025978, https://www.ncbi.nlm.nih.gov/sra/SRX1026041]. The remaining synthetic samples, i.e., DREAM4 and DREAM5, are available upon request through the ICGC Data Access Compliance Office (https://daco.icgc-argo.org/). Sequence data for the gastric cancer training cohort are from a previously published study and available from EGA under accession code EGAD00001000782 [https://ega-archive.org/datasets/EGAD00001000782], the liver cancer training cohort are also from a previously published study and available upon request through Genomic Data Commons (GDC) [https://portal.gdc.cancer.gov/projects/TCGA-LIHC] (instructions to obtain access can be found here: https://gdc.cancer.gov/access-data/obtaining-access-controlled-data). Sequence data for the remaining training cohorts, i.e., sarcoma, lymphoma, colorectal, thyroid and lung, are available upon request due to lack of patient consent to deposit in a repository. Requests for access will be processed within 1 month subject to signing of a data-use agreement (e-mail skanderupamj@gis.a-star.edu.sg), access will be provided for the duration of the project requiring the data. All samples in training cohorts were obtained with written informed consent from patients. The gastric and liver training cohorts were obtained from previously published studies after signing data-use agreements. Source code is described in the Code Availability section. Source data for figures and tables are provided with this paper.

Field-specific reporting

Please select the one below that is the best fit for your research. If you are not sure, read the appropriate sections before making your selection.

☒ Life sciences ☐ Behavioural & social sciences ☐ Ecological, evolutionary & environmental sciences

For a reference copy of the document with all sections, see nature.com/documents/nr-reporting-summary-flat.pdf

Life sciences study design

All studies must disclose on these points even when the disclosure is negative.

Sample size	Available public somatic reference datasets of real and synthetic tumors were used for benchmarking (9 in total). Main determinant of sample size is public availability of established somatic reference datasets.
Data exclusions	No sites were excluded in the evaluations using public benchmark datasets. Training samples were selected using call sets from the SMuRF ensemble-caller.
Replication	All results in the study can be replicated using the provided code (https://github.com/skandlab/VarNet) and publicly available benchmark samples described in Data Availability.
Randomization	The method is benchmarked against existing variant callers on the same benchmark samples.
Blinding	Training and benchmark cohorts were separated in this study.

Reporting for specific materials, systems and methods

We require information from authors about some types of materials, experimental systems and methods used in many studies. Here, indicate whether each material, system or method listed is relevant to your study. If you are not sure if a list item applies to your research, read the appropriate section before selecting a response.

Materials & experimental systems

n/a	Involved in the study
☒	☐ Antibodies
☒	☐ Eukaryotic cell lines
☒	☐ Palaeontology and archaeology
☒	☐ Animals and other organisms
☒	☐ Human research participants
☒	☐ Clinical data
☒	☐ Dual use research of concern

Methods

n/a	Involved in the study
☒	☐ ChIP-seq
☒	☐ Flow cytometry
☒	☐ MRI-based neuroimaging
